# Altruism in nursing from 2012 to 2022: A scoping review

**DOI:** 10.3389/fpsyt.2022.1046991

**Published:** 2022-12-09

**Authors:** Yilin Chen, Caixia Xie, Ping Zheng, Yanli Zeng

**Affiliations:** ^1^School of Medicine, University of Electronic Science and Technology of China, Chengdu, China; ^2^Department of Nursing, Sichuan Academy of Medical Sciences and Sichuan Provincial People's Hospital, Chengdu, China; ^3^School of Nursing, Chengdu University of Traditional Chinese Medicine, Chengdu, China

**Keywords:** altruism, altruistic behaviors, nursing, nurses, healthcare workers, scoping review, pathological altruism

## Abstract

**Background:**

Being a nurse with non-altruistic orientation exists and altruism decline is being challenged as never before, which would be a disaster for medicine if left unnoticed.

**Purpose:**

To describe the meaning of altruism and altruistic behaviors in nursing, and to discuss dilemmas we face today.

**Method:**

Cochrane, PROSPERO, PubMed, Web of Science, CINAHL, Scopus, Embase, ProQuest, and CNKI were searched for original research published in English or Chinese from 2012 to February 2022.

**Results:**

By screening 13 studies came from 12 different countries described altruism and altruistic behavior together were included in. Altruism has been described as value, vocation, or professionalism in nursing which can reflect nurses' compassion, level of expertise, and quality of care. Altruistic nursing care, body donation, financial endowment, volunteering, sharing, benefiting patients maximum, and helping colleagues represented most of the altruistic behaviors in nursing. There is a vacant that not any assessment tool designed for measuring altruism in nurse groups. Interventions from curriculums in class and support of organizations with psychological methods could be helpful to improve the nurses' level of altruism.

**Conclusion:**

Altruism and altruistic behaviors in the past decades were described. A new concept of altruism in nursing was proposed based on the original meaning and the current changes, and interventions for promoting altruism and some of the dilemmas faced today were synthesized.

## Background

Altruism means helping others without the direct or indirect expectation of a reward (1). Its definition is that serves the best interests of the patients. When altruism fits into medicine (2). It is an ethical value that drives nurses' caring behaviors and the choice of nursing as a career, when altruism is further applied in nursing (3). The altruistic behaviors of nurses oriented by empathy or compassion are mainly represented by volunteer activities, organ donation, and money donation, etc. Additionally, altruistic care toward patients that puts the patient's interests first can be the most important type of altruism in nursing work (4).

Altruism is beneficial to nurses' health. Altruism leads to an individual sense of well-being and health, a sense of enjoyment resulting from work, and an association with better longevity (5). Focusing on helping others survive and thrive has benefits for both patients and nurses, and being a helper has a higher correlation with mental health than being the receiver of help (6). On the contrary, not only are they not liked, but they are also less likely to receive help, opportunities to be hired or promoted, fair pay and status, power, and independence in the workplace if nurses' level of altruism decreases (7). Evidence suggests that women always face much more punishment for failing to be altruistic because of gender stereotype-based behavioral prescriptions (8). Most nurses are female and they will usually receive hostility from patients or punishment from the organization if they do not behave altruistically as the public expects (9, 10). Thus, raising the nurses' altruistic levels is worthwhile for both health and social reasons.

Aside from these impacts on nurses, patients can also be affected. A higher altruism level was associated with a better quality of nursing. In addition, nurses responding to the patients' ethical requirements can demonstrate professionalism and let the patient feel more thoroughly cared for (11). Altruistic care plays an important role in the nurse-patient relationship. It helps to build a strong, trusting relationship that can reduce many conflicts. Conversely, when altruistic care decreases, nursing quality also declines. The result is low nursing satisfaction and a high complaint rate it (3). Even worse, the reputations of nurses can be diminished by a lack of altruism, and it can counteract efforts to show respect to nurses (10, 12). From this, it can be seen that altruism helps gain the patients' recognition and compliments.

However, some studies have found that altruism has been decreasing over time (13, 14). With the background of increasing mistrust between the medical profession, the media, and the public and increasing incidents of violence against healthcare professionals, altruism has been decreasing (14). Additionally, altruism in medicine is being threatened by the erosion of empathy in the clinical working environment and the growing rate of burnout (3). Recently, professionalism and wellness have been evoked to respond to this situation. At the same time, calls to abandon altruism in the modern marketplace of medicine also existed (15).

Therefore, it is necessary to identify the meaning of altruism and its specific expressions in nursing to determine what altruism represents and what problems we faced today. Assessment tools for measuring nurses' altruism and the suggestions to promote it are included in this study as well.

## Methods

A scoping review is used to map the size and scope of published literature on a specific topic, synthesize findings and identify gaps. As a methodology, it facilitates information collection from different sources and study designs concerned with various research questions. This scoping review was conducted based on a five-step methodological framework proposed by Arksey and O'Malley (16).

### Stage 1: Identify the research question

This review sought to answer the following research question: (1) What is altruism in nursing? (2) What are the altruistic behaviors in nursing? (3) What assessment tools are available to measure altruism in nursing? (4) What interventions can promote altruism in nursing?

### Stage 2: Identify the relevant studies

We first confirmed that no similar review existed in the Cochrane library and PROSPERO, and then performed a systematic search across eight databases for articles published from 2012 to February 2022 to obtain the latest evidence in this area. The eight databases are as follows: PubMed, Web of Science, Cumulative Index to Nursing and Allied Health Literature (CINAHL), Scopus, Embase, ProQuest, and CNKI. We identified the main concepts, including “nursing/healthcare professionals,” “altruism,” and “altruistic behaviors.” A combination of Medical Subject Headings (MESH)/Emtree terms and free-text terms was used in our search strategy. The different search strings adapted for each database are displayed in Online Supplementary Table S1. Two independent reviewers performed the search process using the same strategies. The reference lists of the included articles were reviewed thoroughly to search for additional studies.

### Stage 3: Study selection

**Figure 1:**
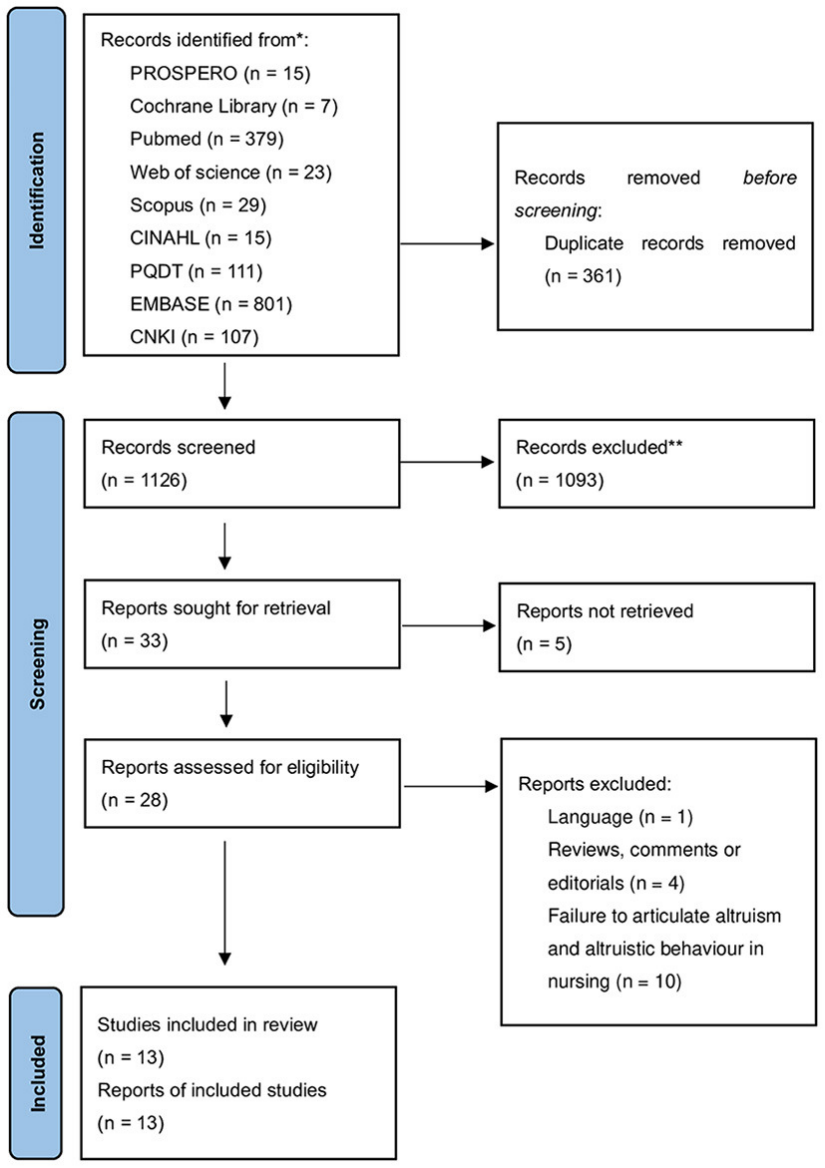
PRISMA-ScR checklist (2018).

Studies were selected based on the inclusion and exclusion criteria. The inclusion criteria were as follows: (a) Published in English or Chinese; (b) elaborating on altruism and altruistic behavior in nursing simultaneously and (c) quantitative study, qualitative study or mixed methods study. Any reviews, comments/opinions, editorials, or study protocols with no empirical data were excluded. Restrictions on the date were made to concentrate on recent clinical practice and to update the previous literature. Two reviewers independently determined the eligibility of studies using a two–step screening process: (1) titles and abstract screening and (2) full text screening. Any discrepancy was resolved by discussion with a third reviewer. The flow diagram is displayed in Figure 1.

### Stage 4: Charting the data

The data were manually extracted from the enrolled studies into Excel and summarized in a tabular format. The extracted data included the following: the first author, year of publication, country, study design, study aims, sample size, participants, altruism in nursing, altruistic behaviors in nursing and assessment tools. Data extraction was performed independently by two investigators for the included literature and discussed with a third researcher in case of disagreement.

### Stage 5: Collating, summarizing and reporting the results

A Table 1 was adopted to synthesize the meaning of altruism, specific altruistic behaviors and related measurements in nursing. In addition, a textual narrative synthesis was adopted to collate the extracted data that did not show up in the table-intervention to improve nurses' altruism. This synthesis was useful in synthesizing evidence of different types (qualitative, quantitative, economic, etc.). The Synthesis Without Meta-analysis guideline (SWiM) was followed (28).

**Table 1:** Data charting table.

**References**	**Country**	**Study design**	**Study aims**	**Sample** **size**	**Participants**	**Altruism in Nursing**	**Altruistic behaviors in nursing**	**Assessment tools**
AlHejaili et al. (17)	Saudi Arabia	Cross-sectional study	To evaluate the awareness of organ transplantation and willingness to donate among Saudi Health Colleges students.	823	Medical students	Value	1. Donating organs after death. 2. Donating organs to a relative/stranger with kidney failure	Customized questionnaire
Bhuvana et al. (13)	India	Cross-sectional study	To assess altruistic attitudes among Medical and Engineering students in a Medical and Engineering College in Bangalore and to determine the factors influencing altruistic behavior among these students	400	Medical and engineering students	Professionalism	voluntary blood donation participation in rural health education activities humanitarian activities in disaster struck areas	The Self-Reported Altruism Scale (SRAS) Rushton et al. (18)
Carter (4)	Australia	Qualitative study	To understand the vocational and altruistic motivations of nurses through the application of Pierre Bourdieu's concepts of “symbolic capital,” “field” and “habitus” through a long interview with nurse respondents.	12	Nurses	Vocation/Value	Making decisions in the interest of the patient without regard to personal loss	The researcher
Ciftci et al. (19)	Turkey	Cross-sectional study	To identify the altruism levels of nursing students and the affecting factors	558	Nursing students	Professionalism	1. Participating in volunteer activities. 2. Helping Financially. 3. Helping in traumatic situations. 4. Caring for the “Elderly/Sick.” 5. Helping based on physical strength. 6. Helping in the education process. 7. Helping from a sense of closeness	Altruism Scale by Ümmet et al. (20)
Hollup (21)	Mauritius	Qualitative study	To describe and analyze those factors and conditions influencing the decision to choose nursing as a career among men and women nurses.	43	Nurses	Vocation/value	Volunteering	The researcher
Kubsch et al. (22)	USA	Mixed-methods study	To study how the stress of caring for difficult patients affects the level of altruism and use of negative coping strategies in their care and to find out what positive coping strategies and interventions could be used.	67	Nurses	Value	1. Acknowledges acts of kindness 2. Honors own and others' gifts and talents. 3. Recognize vulnerabilities in self and others. 4. Treats self and others with loving kindness. 5. Listens respectively with genuine concern to others. 6. Accepts self and others as they are. 7. Demonstrates respect for self and others. 8. Listens to others. 9. Treats others with kindness. 10. Pays attention to others. 11. Respects others. 12. Honors human dignity of self and others. 13. Practice equanimity with patients. 14. Open to connectedness with self, others, environment, universe. 15. Models self-care and caring for others. 16. Validates uniqueness of self and others	Customized questionnaire
Milaniak et al. (23)	Poland	Cross-sectional study	To examine the role of empathy and altruism in organ donation decision making process among nursing and paramedic students.	111	Nursing and paramedic students	Motivation to guide practice	Organ donation	Questionnaire A-N (Altruism- Non-altruism). (by J. Sliwak)
Nasrabadi et al. (24)	Iran	Qualitative study	To understand the Iranian nurses'experiences in the field of job satisfaction.	10	Nurses	The foundation for their job satisfaction	1. Helping patients when they sincerely need help 2. Be an advocate for patients	The researcher
Sanjai et al. (2)	India	Cross-sectional study	To assess altruistic attitudes among medical students in a medical college in Chennai and determining the factors which influence altruism.	224	Medical students	An important virtue	1. Blood donation 2. Organ donation. 3. Participation in clinical trials	The Self-Reported Altruism Scale (SRAS). Rushton et al. (18)
								
Slettmyr et al. (14)	Sweden	Qualitative study	To explore how nurses perceive the meaning of altruism in today's healthcare.	13	Nurses	Vocation/Value	Doing “the little extra” for the patient, that is, going further and doing something that was not expected of them in their daily work.	The researcher
Smith et al. (25)	Kenya, South Africa and Thailand	Cohort study	To characterize the nature and determinants of nurses' altruism, based on a cross-country quantitative study utilizing novel experimental economic field experiments with nursing students in three Low–and middle-income countries (LMICs).	1,064	Nursing students	Value	Financial endowment	Dictator game^†^
Jun et al. (26)	China	Qualitative study	To probe into the attitude of 90's practical nursing students to altruism and its influencing factors.	7	Practical nursing students	Value	Helping each other take care of the patients they manage separately	The researcher
Zhang et al. (27)	China	Cross-sectional study	To understand baccalaureate nursing students′ humanistic care ability, and explore its relationship with altruistic behavior, so as to provide reference for promoting students′ humanistic care consciousness and ability.	437	Nursing students	Professionalism	Helping others without compensation and creating happiness and feeling joy in the process of practicing sharing, humility and helping others for pleasure. Such as: 1. volunteering 2. Giving up one's seat 3. Donating money	Altruistic Behavior Questionnaire for College Students by Zhang et al. (27)

† A “dictator game” (DG) was a standard technique within experimental economics for detecting the presence and power of altruism in decision-making.

## Results

### Search outcomes and study characteristics

A PRISMA flow diagram illustrating the search process is shown in Figure 1. A total of 1,487 references were searched in 9 databases. Among these, 361 duplicates were removed, and 1,093 articles were excluded after screening the titles and abstracts. For five articles, we did not find the full text. A total of 28 full-text papers were assessed and screened for eligibility. One Russian article and one German article were excluded for linguistic reasons. Five articles were excluded due to their literature type, as they were reviews, editorials, or comments. In addition, five studies addressed only altruistic values and did not elaborate on altruistic behaviors in nursing. Ultimately, 13 studies were included to shed light on altruism and altruistic behavior in nursing.

Details of the study characteristics are provided in Table 1. The 13 studies came from 12 different countries: Saudi Arabia (*n* = 1) (17), India (*n* = 2) (2, 13), Australia (*n* = 1) (4), Turkey (*n* = 1) (19), Mauritius (*n* = 1) (21), USA (*n* = 1) (22), Poland (*n* = 1) (23), Iran (*n* = 1) (24), Sweden (*n* = 1) (14), Kenya (*n* = 1), South Africa (*n* = 1), Thailand (*n* = 1) (25) and China (*n* = 2) (26, 27). The year of publication ranged from 2012–2021. The types of studies included cross-sectional studies (*n* = 5), qualitative studies (*n* = 5), a cohort study (*n* = 1), and a mixed study (*n* = 1). No studies involved interventions, and all were classified as observational studies. A population of 3,332 participants was included in this review. The sample size in each study ranged from 7 to 1,064. Most participants were nursing students and nurses were included in qualitative studies only. This meant that most of the research was conducted in schools rather than in hospitals.

### Altruism in nursing

Altruism was described as a value, vocation or aspect of professionalism in nursing. It was the core of the nursing profession or the basis for choosing nursing as a profession. More than half of the studies identified altruism as a personal or work value (4, 14, 17, 21, 22, 25, 26). Three studies perceived it as the professional equivalent to professional skills and as a reflection and representation of the level of expertise (13, 19, 27). Another three studies treated altruism as the value of the vocation that participants adopted not to earn a living but because of self-perceived responsibility and self-esteem realization (4, 14, 21). One study identified altruism as a motivation to guide practice and one study considered it as the foundation for job satisfaction (23, 24).

### Altruistic behaviors in nursing

The sixteen competencies of altruistic nursing care are proposed based on Watson's Theory of Human Caring, and the details were as follows: (1) acknowledges acts of kindness; (2) honors own and others' gifts and talents; (3) recognizes vulnerabilities in self and others; (4) treats self and others with loving kindness; (5) listens respectively with genuine concern to others; (6) accepts self and others as they are; (7) demonstrates respect for self and others; (8) listens to others; (9) treats others with kindness; (10) pays attention to others; (11) respects others; (12) honors human dignity of self and others; (13) practices equanimity with patients; (14) is open to connectedness with self, others, environment, and the universe; (15) models self-care and caring for others; and (16) validates the uniqueness of self and others (22, 29). Organ donation was mentioned in three studies, including living donation and post-mortem donation; living donation mainly refers to donating organs to a relative/stranger with kidney failure (17). Blood donation was cited in two studies (2, 13) and financial endowment was addressed in three studies (19, 25, 27). Volunteering was noted in four studies, including teaching in remote places, participating in clinical trials, and taking part in various volunteer activities (13, 19, 21, 27). Making decisions in the interest of the patient without regard to personal loss (4) and doing “a little extra” for the patient were also be mentioned (14). In addition, sharing knowledges among the colleagues (21) and helping each other take care of the patients they manage separately were described as well (26).

### Assessment tools for altruism in nursing

The Self-Reported Altruism Scale (SRAS) developed by Rushton in 1981 was used in two studies to measure the altruism of medical students included nursing students (2, 30). It consisted of 20 items and used 5-Point Likert Scale, and showed good reliability (Cronbach' s α = 0.89) and high degree of validity (r = 0.78) (13). Moreover, it was designed for students from universities originally and then widely used in public.

The Altruism Non-altruism (A-N) Questionnaire designed by J. Sliwak was applied to one study to assess altruism in nursing and paramedic students (23). The Questionnaire A–N was designed for the public which contains 10 stories, each with 6 answers provided to reflect various degrees of intensity of the altruistic attitude. It has good reliability and validity (Cronbach' s α = 0.87, r = 0.86) but used limited because the original scale was Polish (31).

The Altruistic Behavior Questionnaire for College Students created by Li Yanfang in 2010 was utilized for altruistic behaviors among nursing students (10). It adopted 7-Point Likert Scale which contained 30 items and showed good reliability and validity (Cronbach' s α = 0.873, r = 0.739–0.789) (32). And it was also limited because of language and designed population.

A well-established method in experimental economics called the “Dictator Game” (DG) was adopted to detect the presence and power of altruism in the decision-making of nursing students (25, 33).

The rest of the studies used a customized questionnaire or selected interviews as their way to survey altruism or altruistic behaviors in nursing students or nurses. It can be inferred that there is some bias in measuring nurses' altruism due to the inconsistent measurement tools. Moreover, the self-reported scales or questionaries could not reflect reality appropriately because of the effect of social approval, self-recognition, and cultural background, etc. Meanwhile, we did not find any assessment tools designed for nurse groups, which means that tools may be unable to investigate altruistic behaviors in the nursing workplace.

## Discussion

The objective of this scoping review was to identify the meaning of altruism and its specific altruistic behaviors in nursing and to determine what kinds of dilemmas we face today. Although the included studies addressed the questions advanced, they were still limited.

### Meaning of altruism

To sum up, considering that core of altruism is to help others without reward, the recent decline in the will to sacrifice, pathological altruism and the high rate of burnout and compassion fatigue are concerning. This paper defines altruism in nursing as putting the patient's interests first, focusing on others rather than being other-driven, trying to meet the patient's ethical needs and providing altruistic care without exhausting compassion while taking care of one's feelings, balancing helping others with self-sacrifice and not being overly critical of oneself if one fails to do one's best. Most of the included studies described altruism as a personal value, work value, aspect of professionalism, or vocation. Although altruism is not as highly valued as other professional skills and is frequently dismissed, it is equal to the professional knowledge and skills that can manifest a level of expertise. This is consistent with a previous study showing that professionalism is a core competency that requires lifelong learning, commitment, and practice and can be influenced by character (34). The help and encouragement from administrators or organizations could be helpful on the road to professionalism. In addition, altruism was treated as the foundation for job satisfaction. Altruism was described as the main essence of nurses' job satisfaction, which includes the three aspects of patient advocacy, spiritual job satisfaction, and professional commitment (24). Nurses have a pleasant feeling along with enjoyment resulting from addressing the needs of patients (24). Providing care to patients with all their love amidst the many difficulties of their professions can stimulate nurses' favorable emotional experiences and motivate their desire to practice in clinical work (23). We can infer that altruism still plays an important role as motivation in nursing work.

### Faded altruistic behaviors

In this review, we categorized the altruistic behavior of nurses as follows: (1) volunteering in education, social activities, humanitarian activities, or clinical trials; (2) donating physically, such as blood, living organs, or remains; (3) financial endowment; (4) sharing knowledge; (5) doing ‘a little extra' for patients that were not expected of them in their daily work; (6) making decisions in the interest of the patient and reducing unnecessary medical treatment; (7) helping each other take care of the patients they manage separately. Bhuvana et al. (13) and Sanjai et al. (2) found that the frequency of altruistic behavior among medical students was low, and most showed simple only altruistic behaviors. Even worse, calls to abandon altruism in the modern marketplace of medicine also existed (15). This may be because of the resistance to self-sacrifice and burnout. A reasonable distribution of nursing staff and a reduction of workload may help.

### Limited measurement

There were three measurement methods used to evaluate altruism or altruistic behaviors generally: (1) scales, (2) situational stimulated methods, and (3) the experimental Economics Measurement Method. The assessment tools applied to the 13 studies were inconsistent. Except for the qualitative study, the Self-Reported Altruism Scale (SRAS) developed by Rushton was frequently used, which is also called the altruism personality scale. The SRAS is widely used as a relatively authoritative scale to measure altruism in the public. In addition, the Self-Reported Altruism Scale (SRAS-DR) developed by Ryo Oda in 2013 (35), the Philosophical Moral-Framing Measure (PMFM) developed by Julian Friedland in 2020 (36), The Altruism Scale for Adults developed by Lee DY in 2003 (37), the Scale of Attitude toward the Patient (SatP) developed by Jakub Pawlikowski in 2012 (38) the Philosophies of Human Nature Scale (RPHN Life Scale) developed by Wrightsman (39) and customized questionnaires have been used with nursing students, nurses or physicians as well in previous research. From this, we can infer that the measurement is too homogeneous and limited to scales or questionnaires, and there are no scales designed for nurses' groups. And the universal measurements designed for the public can not reflect the attitudes or behaviors among patients or nursing workers appropriately.

### Intervention to improve nurses' altruism

We found that the included studies were cross-sectional studies, qualitative studies or mix-methods studies and none of them were designed with intervention to improve nurses' altruism. However, we also found compassion was a valuable point of focus which could be stimulated by compassion or empathy (40, 41). Compassion is practiced through a variety of experiential practices and meditations. In addition to showing compassion to others, increasing self-compassion and reducing self-criticism and self-persecution are equivalently helpful (42). Bhuvana and colleagues proposed three improvements: (1) the enhancement of emotional intelligence and professional ethics and values; (2) skills training for empathy, medical-patient communication, and good medical-patient relationship building; (3) special teaching programmes such as problem-based learning (PBL), Case-Based Learning (CBL) and the Attitude and Communication Module (AETCOM), including ethics-related courses and training. Carter proposed two improvements: (1) Pierre Bourdieu's theory of practice has proved useful as a method to understand the complexities of nursing motivations in contemporary society; (2) workforce planning, workplace culture, nurse recruitment, and nurse education (4). Gebriné suggested creating a work environment that is physically conducive and inspiring in its layout, as well as intellectually and emotionally stimulating (43). Kubsch proposed two improvements: (1) developing Psychological Hardiness to remain resilient by employing several complementary therapies, such as deep breathing, walking, and yoga; (2) bearing witness to understand the needs and stories behind the patients when facing difficult patients. The difficult patient may be a survivor of mental or physical trauma, sexual abuse, homelessness, abandonment, and so on (22). Sanjai proposed five improvements: (1) a curriculum for medical professionalism (i.e., a medical professionalism curriculum should emphasize skills such as communication, empathy, emotional intelligence, and professional values in addition to preparing students for a lifelong self-directed career and the pursuit of professional excellence); (2) the influence of parents, peers and role models; (3) volunteering; (4) participation in medical camps; and (5) humanitarian activities. In summary, altruism as a work value is a requirement for work; it should be cultivated through school learning and ongoing work. It may be consistent with personal values and determine one's attitude and behaviors or it may not (44, 45).

### The dilemma of altruism in nursing

#### Altruism can be diminished

It was found that altruism has eroded over time and is not as highly valued today as it was in the past (14, 22). This may be because, in our traditional setting, medical students are subjected to a high level of stress, a great deal of information, uncertain scenarios, high levels of responsibility, and intense competition as a result of their clinical training and the possibility of being threatened at any time. In this atmosphere, medical students undergo a change known as “traumatic de-idealization,” and maintaining the ideal trait of altruism is difficult (13). Apart from that, the emphasis on individualism conflicts with self-sacrifice. Since medicine adopted the consumer-provider model, with more emphasis on performance allocation, altruistic behaviors could have come into conflict with the schema of individual vs. collective interests constantly, and the role of altruism has become vague (2, 13). A sense of alienation from patients and burnout can develop and empathy can diminish when nurses are faced with an increasingly heavy workload and the endless demands of patients. Self-protection by treating patients in an objective, technical, detached, and non-humanistic manner is often seen in medical culture (22).

#### Altruism can be isolated from nurses' work

Previous studies have argued that people choose to pursue a nursing career because they are altruistically oriented, desire to work and help others or consider nursing a meaningful profession; often, it is the first and only educational choice (46). However, as recent studies in the Nordic countries have shown, an increasing number of students are entering nursing education not because they love nursing but because they are not given the course of study they prefer, or lack other opportunities (47). Most nursing practitioners consider nursing to be a way to earn a living, do not see it as different from other jobs, and are not motivated by an altruistic view of the profession or a sense of having a mission (21). Work values may be consistent with personal values and determine one's attitude and behaviors or not (44, 45). Inconsistency between individual values and work values can exit occasionally, and attitudes toward or behaviors at work can be different from those in individual life (2, 44, 45). Thus, one can be an altruist in life but not in the working environment. The point we need to focus on is letting altruism emerge both in personal and work values. Work values can be enhanced by education in hospital culture, rendering of departmental atmosphere, and guidance of role models. Value maintenance in subsequent practice can be treated as professionalism, which is also required by clinical work.

#### Altruism can be exploitative or unethical

There are ethical dilemmas such as end-of-life care, physician conflicts, organizational constraints, family conflicts, and privacy and dignity. In end-of-life care and physician conflicts, the point is always futile treatments or over treatments. The obligation of “Advocating for the patient” may conflict with the principle “Do not harm” when death is the better option for patients rather than futile treatment or over treatments. As for organizational constraints, staffing shortages and poverty can lead to failure to give the best care to patients. Regarding family conflicts, it was a challenge to provide critical care when patients' claims were inconsistent with those of their families and the patient was not the decision maker. There can also be a dilemma with the priority of protecting privacy and dignity or safety when patients reject nurses' help but also need that help (48). For example, this may occur when patients go to the toilet by themselves, with a high risk of falling. Moreover, some altruistic behaviors, such as surrogacy, can be unethical or even illegal (49). However, we cannot always prevent such behaviors from happening due to diverse cultures or religions.

#### Pathological altruism

Pathological altruism can be prevalent among some healthcare professionals, with negative consequences for healthcare professionals and patients. Pathological altruism is any behavior with the motivation to promote the welfare of another that, instead of beneficial outcomes, leads to negative consequences for the other or even for the self. The causes of pathological altruism are as follows: fear of humiliation, an unconscious need for social approval, a compulsion to fix, save and help others; a sense of conviction that one's actions are both morally correct and serve an ultimate good; strict adherence to religious rules; empathy-based guilt; and unhealthy power dynamics all contribute to harmful altruism (50). In addition, a new phenomenon emerged in which vocation has become associated with negative phrases, such as sacrifice, obedience, and submission, and is perceived in modern discourse as the antithesis of professionalism and paid work (51). We found that fewer studies were focused on pathological altruism in medicine and none of them were in nursing. This may be related to the traditional views, which treat altruism as part of medicine already and ignore the changes.

#### Commerciality in altruism

Most people in the nursing profession cannot ignore the need to earn a living and can be in an awkward position relative to other health disciplines when it comes to the financial rewards associated with their contributions to health care. Apart from individual commerciality, changes in the whole industry need to be mentioned. Medicine has become increasingly accountable, and healthcare professionals are subject to business drivers and performance metrics (52). This may be due to a consumer-provider model taking shape in medicine in recent years. In this case, there has been a growing sense of declining altruism in medicine as medicine has become more focused on evaluating the costs and benefits of the medical profession and the increasing number of medical malpractice cases (2).

#### Motivation for becoming a nurse can be non-altruistic

Altruistic tendencies are high among those who choose a nursing career, and vice versa. However, motivation for becoming a nurse may be non-altruistic. In some less economically developed regions, a career in nursing has become a better choice for international migrants as a springboard to cross classes (49). Between 1990 and 2007, the number of nurse recruiting firms in the United States grew ten-fold, and the number has been increasing persistently (53, 54). As developed countries enter an aging society, the demand for caregivers has been increasing, and the higher remuneration for the job, the better employment environment, and the opportunity for continued good education correspondingly will inevitably attract potential nurses to choose international migration. This could exacerbate the shortage of nurses in developing countries. Therefore, listening to nurses' claims about clinical work and making adjustments according to the promotion of nurses' salary, working environment, and education opportunities is urgent.

### Challenges in the development of nursing discipline construction

In the past, nursing was an applied science that pull the theoretical knowledge from a range of natural (biology, chemistry, physics, and others) and social sciences (sociology, psychology, ecology, economics, anthropology, epidemiology, and others) and attached to clinical medicine. But now, a transition from vocational training to higher-level education which leads to the nursing discipline getting diverse, scientific, and academic. There is a continuous increase in the number of highly educated nurses with the nursing education level becoming more and more well-established and consummate (30). Nurses can also fight for their rights and make conscientious objections because of the development of nursing education, nursing science, and evidence-based nursing practice. The structure and form of nursing science depend on the nature of its unique knowledge base, rather than the various activities that nurses are engaged in and different from clinical medicine. To be sure, nursing is more extensive than its science including the philosophy of nursing, nursing history, nursing education, and other fields (55). Thus, nursing disciplines should have the voices of nurse philosophers, nurse historians, nurse educators, nurse scientists, nurse practitioners, and other correlative types. We need to focus on the ontologies of nursing which mean solidly anchored in nursing instead of blindly engaging in meaningless interdisciplinary studies. And utilizing knowledge through organizations, leaders, educators, researchers, etc. to identify new values for nursing in the new society and let these contemporary evolutions compatible with altruism. In a word, both investigations or studies deeply rooted in nursing by researchers, support and respect from organizations, and the discourse power of nursing leaders, etc. can contributeto the construction of the nursing discipline. And it is also a persistent problem that needs to be handled indeed.

## Strengths and limitations

This scoping review followed the PRISMA-ScR checklist and adopted the rigorous methodology developed by Arksey and O'Malley. Nine databases were searched for the study, including PROSPERO, Cochrane Library, Pubmed, Web of Science, Scopus, CINAHL, PQDT, EMBASE, and CNKI. The research questions were identified in this study and information was found beyond them.

There were four limitations to the study: (1) unregistered; (2) insufficient articles that covered altruism and altruistic behaviors concurrently; (3) languages except English or Chinese were not included and (4) the studies involved in this review did not get any quality assessment, since the scoping review did not have strict rules requiring quality evaluation before inclusion in the literature.

## Conclusions

A definition of altruism and altruistic behaviors in nursing has been proposed. The dilemma we faced today and some interventions were also described in this article. Moreover, based on this study, there are three points worthy of further explanation: (1) scales designed for altruism in nursing; (2) research to investigate altruistic behaviors in daily nurses' work for patients in the future and (3) studies focused on pathological altruism in medicine.

Apart from that, we call for less advocacy of self-sacrifice in an area that values the development of individualism to ease nurses' burnout of being altruistic all the time and the guilty of not altruistic enough. Additionally, it should be mentioned that nurses' requirements for safety, wages, education and respects, etc.

## Author contributions

YC: conceptualization, methodology, investigation, data curation, writing—original draft, and visualization. CX: writing—review and editing and supervision. PZ: data curation, visualization, writing—review and editing. YZ: writing—review and editing. All authors contributed to the article and approved the submitted version.
