# Supplementary material for: Altruism in nursing from 2012 to 2022: A scoping review
Source: Front Psychiatry. 2022 Dec 9;13:1046991. doi: 10.3389/fpsyt.2022.1046991 (PMC9780546; doi:10.3389/fpsyt.2022.1046991)
Supplement: Supplementary file 1 [file Data_Sheet_1.doc]

**Supplementary File 1 Search strategies of each database (2012-2022)**

| Database | Search Strategy | Literature |
| --- | --- | --- |
| PROSPERO | #1 MeSH DESCRIPTOR Nursing EXPLODE ALL TREES  #2 MeSH DESCRIPTOR Nurses EXPLODE ALL TREES  #3 MeSH DESCRIPTOR Physicians EXPLODE ALL TREES  #4 MeSH DESCRIPTOR Health Personnel EXPLODE ALL TREES  #5 nurs* OR midwi* OR nurse practitioners OR doctor* OR clinician* OR physician* OR healthcare workers OR healthcare professionals OR healthcare providers OR health personnel OR medical staff OR medical practitioner OR medical care personnel  #6 #1 OR #2 OR #3 OR #4 OR #5  #7 MeSH DESCRIPTOR Altruism EXPLODE ALL TREES  #8 altruism OR altruistic behavior  #9 #7 OR #8  #10 #6 AND #9  #11 * WHERE CD FROM 01/01/2012 TO 1/02/2022  #12 #10 AND #11 | 15 |
| Cochrane Library | #1 MeSH descriptor: [Nursing] explode all trees  #2 MeSH descriptor: [Nurses] explode all trees  #3 MeSH descriptor: [Nurse Midwives] explode all trees  #4 MeSH descriptor: [Nurse Practitioners] explode all trees  #5 MeSH descriptor: [Health Personnel] explode all trees  #6 (nurs* OR midwi* OR nurse practitioners OR doctor* OR clinician* OR physician* OR healthcare workers OR healthcare professionals OR healthcare providers OR health personnel OR medical staff OR medical practitioner OR medical care personnel):ti,ab,kw with Cochrane Library publication date Between Jan 2012 and Feb 2022, in Cochrane Reviews, Trials  #7 {OR #1-#6}  #8 MeSH descriptor: [Altruism] explode all trees  #9 {AND #7, #8} | 7 |
| Pubmed | 1 " Nursing " [MeSH Terms]  2 " Nurses " [MeSH Terms]  3 " Nurse practitioners " [MeSH Terms]  4 " Nurse midwives " [MeSH Terms]  5 " Health Personnel "[MeSH Terms]  6 " physicians " [MeSH Terms]  7 #1 OR #2 OR #3 OR #4 OR #5 OR #6  8 ((((((((((((((nurs*[Title/Abstract])) OR (healthcare professional[Title/Abstract])) OR (midwi* [Title] OR [Title])) OR (nurse practitioners[Title/Abstract])) OR ( doctor*[Title/Abstract])) OR (clinician*[Title/Abstract])) OR (physician*[Title/Abstract])) OR (healthcare workers[Title/Abstract])) OR (healthcare professionals[Title/Abstract])) OR (healthcare providers[Title/Abstract])) OR (health personnel[Title/Abstract])) OR (medical staff[Title/Abstract])) OR (medical practitioner[Title/Abstract])) OR (medical care personnel[Title/Abstract])  9 #7 OR #8  10 "altruism"[MeSH Terms]  11 ((altruism[Title/Abstract])) OR (altruistic behavior*[Title/Abstract])  12 #10 OR #11  13 #9 AND #12 | 379 |
| Web of Science | 1 TI=(nurs* OR midwi* OR nurse practitioners OR doctor* OR clinician* OR physician* OR healthcare workers OR healthcare professionals OR healthcare providers OR health personnel OR medical staff OR medical practitioner OR medical care personnel)  2 TI=(altruism OR altruistic behavior)  3 #1 AND #2 | 23 |
| Scopus | TITLE ( nurs* OR midwi* OR {nurse practitioners} OR doctor* OR clinician* OR physician* OR {healthcare workers} OR {healthcare professionals} OR {healthcare providers} OR {health personnel} OR {medical staff} OR {medical practitioner} OR {medical care personnel} ) AND TITLE ( altruism OR {altruistic behavior*} ) AND ( LIMIT-TO ( PUBYEAR , 2021 ) OR LIMIT-TO ( PUBYEAR , 2020 ) OR LIMIT-TO ( PUBYEAR , 2019 ) OR LIMIT-TO ( PUBYEAR , 2018 ) OR LIMIT-TO ( PUBYEAR , 2017 ) OR LIMIT-TO ( PUBYEAR , 2016 ) OR LIMIT-TO ( PUBYEAR , 2014 ) OR LIMIT-TO ( PUBYEAR , 2013 ) OR LIMIT-TO ( PUBYEAR , 2012 ) ) | 29 |
| CINAHL | S1 TI ( nurs* OR midwi* OR nurse practitioners OR doctor* OR clinician* OR physician* OR healthcare workers OR healthcare professionals OR healthcare providers OR health personnel OR medical staff OR medical practitioner OR medical care personnel )  S2 TI ( altruism OR altruistic behavior )  S3 DT 2012-2022  S4 #S1 AND #S2 AND S3 | 15 |
| PQDT | (Title: nursing OR nurse OR physician OR docotor OR clinician OR healthcare provider OR healthcare worker) AND (Title: altruism or altruistic behavior) AND (Year of publish: 2012-2022) | 111 |
| EMBASE | 1 (nurs* or midwi* or nurse practitioners or doctor* or clinician* or physician* or healthcare workers or healthcare professionals or healthcare providers or health personnel or medical staff or medical practitioner or medical care personnel).mp. [mp=title, abstract, heading word, drug trade name, original title, device manufacturer, drug manufacturer, device trade name, keyword heading word, floating subheading word, candidate term word]  2 (altruism or altruistic behavior).mp. [mp=title, abstract, heading word, drug trade name, original title, device manufacturer, drug manufacturer, device trade name, keyword heading word, floating subheading word, candidate term word]  3 limit 1 to yr="2012 - 2022"  4 1 and 2 and 3 | 801 |
| CNKI | 1 Theme=nursing + midwives + nurse practitioners + doctors + clinicians + physicians + healthcare workers + healthcare professionals  2 Theme=altruism + altruistic behavior  3 #1 AND #2 | 107 |
